# Daily life after the death of a loved one: A systematic review of daily diary and ecological momentary assessment studies

**DOI:** 10.1016/j.ijchp.2026.100693

**Published:** 2026-06-04

**Authors:** Turan Deniz Ergun, Mustafa Anil Topal, Peter M. ten Klooster, Gerben J. Westerhof, Ernst T. Bohlmeijer, Lonneke I.M. Lenferink

**Affiliations:** a Department of Psychology, Health & Technology, Faculty of Behavioural Management and Social Sciences, University of Twente, P.O. Box 217, 7500 AE, Enschede, the Netherlands; b Department of Psychology, University of Toronto, 100St George Street, Toronto, ON, M5S3G3, Canada; c Department of Clinical Psychology and Experimental Psychopathology, Faculty of Behavioral and Social Sciences, University of Groningen, Grote Kruisstraat 2/1, 9712 TS, Groningen, the Netherlands

**Keywords:** Grief, Loss, Experience sampling, Diary, Daily

## Abstract

The death of a loved one is a common yet potentially traumatic event that can impact bereaved people’s daily lives substantially. However, bereavement experiences have been investigated mostly with cross-sectional surveys, which provide limited information about bereaved people’s everyday functioning. Intensive longitudinal methods (ILM) that collect self-reports once (daily diary; DD) or multiple times (ecological momentary assessment; EMA) a day allow researchers to examine momentary variations in bereaved people’s everyday experiences. In this preregistered study, we aimed to systematically review studies that investigated the daily life of bereaved people using ILM. We searched for English peer-reviewed journal articles in Scopus, PubMed, and PsycINFO. After title, abstract, and full-text screening of 230 records, 18 articles were included in the final review. Fourteen studies used DD, and four used EMA designs. Studies examined four topics: a) the acceptability, feasibility, and reactivity effects of ILM among bereaved people, b) emotions and emotion regulation strategies, c) sleep quality, and d) romantic relationships. While the acceptability, feasibility, and reactivity effects of ILM were investigated by both DD and EMA studies, the latter three topics were examined only by DD studies. The studies show that DD and EMA are acceptable and feasible methods among bereaved people. DD studies showed that experiences of positive emotions and affectionate touch were related to better psychological outcomes, such as intimacy, while there were inconsistent associations between grief and sleep quality. ILMs appear to offer valuable yet largely untapped potential for capturing dynamic experiences of grief in bereavement research.

## Introduction

The death of a loved one is one of the most common yet potentially traumatic events that can happen to people (Benjet et al., 2016; Kessler et al., 2017). While there is an increasing body of research on psychological, social, and physical functioning following bereavement (Ennis & Majid, 2021; Kristensen et al., 2012; Lancel et al., 2020; Stroebe et al., 2007), most studies used cross-sectional designs. In these studies, people are typically asked about the impact of the loss on their lives retrospectively (e.g., in the past week or month). However, these retrospective accounts provide limited information about real-time everyday experiences and the contexts in which they occur. Additionally, retrospective evaluation can lead to error and bias in recalling and reporting events and experiences (Shiffman et al., 2008). For instance, previous studies have indicated that people can overestimate or underestimate the intensity of experiences when thinking about the past (Ben-Zeev & Young, 2010; Ellison et al., 2020; Urban et al., 2018).

One way to overcome the limitations of cross-sectional research is to use an intensive longitudinal method (ILM). ILMs can be defined as data collection methods in which people are asked to complete measurements on multiple occasions (Bolger & Laurenceau, 2013; Mehl & Conner, 2012; Myin-Germeys & Kuppens, 2022), such as daily diary (DD) and ecological momentary assessment (EMA; also called experience sampling methodology) designs. In DD designs, people are asked to report on their experiences and/or behaviours only once a day for multiple days (Schneider et al., 2020), while EMA designs include multiple measurements per day across multiple days (Myin-Germeys et al., 2018; Myin-Germeys & Kuppens, 2022). ILMs allow for assessing the momentary health impact(s) of the death of a loved one in a more ecologically valid manner (Hruska et al., 2025) and enable researchers to investigate both the dynamics within individuals and differences between people (Bolger & Laurenceau, 2013; Myin-Germeys et al., 2009). Hence, ILM studies could provide a valuable opportunity to examine the psychological, social, and physical functioning following the death of a loved one in greater detail within the context of bereaved people’s daily lives.

The reactions to the death of a loved one are theorized to be dynamic, fluctuating within bereaved people’s everyday lives (Stroebe & Schut, 1999). In addition, considering that daily life is context-dependent and people encounter different types of stressors in their daily life, bereaved people are likely to vary, both within themselves and among each other, in their responses to and coping with bereavement (Ryckebosch-Dayez et al., 2016). Despite the relevance of examining daily life experiences of bereaved people, to our knowledge, no studies have synthesized what previous research has revealed about the daily life experiences after the death of a loved one in a systematic manner. As the number of ILM studies in health and psychology research has been growing rapidly throughout the years (Fritz et al., 2024; Hamaker & Wichers, 2017), providing a review of studies examining the psychological, social, and physical functioning following the death of a loved one is timely. Moreover, understanding to what extent and how ILMs are used in bereavement research, and what research questions these studies have tried to answer, still remains an important research gap in the literature.

Therefore, we conducted a systematic review of DD and EMA studies that examined daily psychological, social, and/or physical functioning in bereaved people. The main objectives were to a) identify and present an overview of the methods, characteristics, and quality reporting of ILM studies on the impact of the death of a loved one on psychological, social, and physical functioning, b) provide a narrative synthesis of their findings with respect to daily psychological, social, and physical functioning, and c) discuss some of the commonalities across reviewed studies and formulate directions for future ILM research in the field of bereavement.

## Method

The Preferred Reporting Items for Systematic Reviews and Meta-Analyses 2020 (PRISMA; Page et al., 2021) and its Literature Search Extension (PRISMA-S; Rethlefsen et al., 2021) were followed in this systematic review. We pre-registered our search protocol and rationale with the International Prospective Register of Systematic Reviews (PROSPERO; registration number: CRD42024553192).

### Search strategy

We conducted our systematic search in May 2025 using three scientific databases: Scopus, PubMed, and PsycINFO. Two topics structured the search terms, namely a) ILM (i.e., DD and EMA), and b) the death of a loved one. We selected the search terms based on previous systematic reviews conducted on ILM studies (Vaessen et al., 2021) and the death of a loved one (Lancel et al., 2020). We did not include any search terms related to psychological, social, or physical functioning because we did not want to limit our search to certain specific reactions (e.g., grief, depression). The full search strategy for each database is presented in Table 1.

**Table 1:** Search strings.

Database	Search string
PsycINFO	TI ("bereaved" OR "bereavement" OR "grie*" OR "grieving" OR "mourn*" OR "death of a loved one")) AND TI ( "ambulatory monitoring" OR "ambulatory assessment" OR "experience sampling" OR momentary OR diary OR diaries) OR AB "bereaved" OR "bereavement" OR "grie*" OR "grieving" OR "mourn*" OR "death of a loved one")) AND AB ("ambulatory monitoring" OR "ambulatory assessment" OR "experience sampling" OR momentary OR diary OR diaries) OR KW ("bereaved" OR "bereavement" OR "grie*" OR "grieving" OR "mourn*" OR "death of a loved one")) AND KW ("ambulatory monitoring" OR "ambulatory assessment" OR "experience sampling" OR momentary OR diary OR diaries)
Pubmed	((("bereav*" [Title/Abstract] OR "bereavement" [Title/Abstract] OR "grie*" [Title/Abstract] OR "grieving" [Title/Abstract] OR "mourn*" [Title/Abstract] OR "death of a loved one" [Title/Abstract]) AND ("ambulatory monitoring" [Title/Abstract] OR "ambulatory assessment" [Title/Abstract] OR "experience sampling" [Title/Abstract] OR momentary [Title/Abstract] OR diary [Title/Abstract] OR diaries [Title/Abstract])))
Scopus	TITLE-ABS-KEY(((("bereav*" OR "bereavement" OR "grie*" OR "grieving " OR "mourn*" OR "death of a loved one") AND ("ambulatory monitoring" OR "ambulatory assessment" OR "experience sampling" OR momentary OR diary OR diaries))) AND (LIMIT-TO (DOCTYPE,"ar")) AND (LIMIT-TO (LANGUAGE,"English ")))

### Inclusion and exclusion criteria

Quantitative peer-reviewed journal articles in English were eligible to be included. We included any observational study where participants reported their functioning after the death of a loved once a day (i.e., DD) or multiple times a day (i.e., EMA) for at least seven consecutive days. We did not set any exclusion criteria based on death-related factors, such as the cause of death, kinship to the deceased, and time passed since the death. Any intervention studies that used EMA (e.g., ecological momentary interventions) were excluded.

### Study selection

**Fig. 1:**
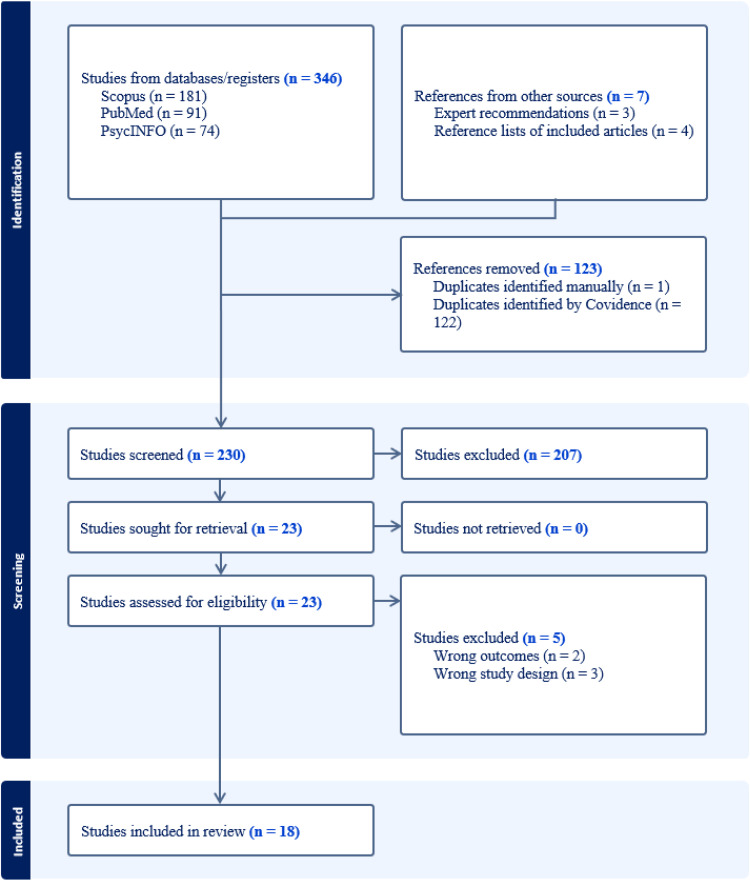
PRISMA flow chart.

Two researchers (TDE and MAT) independently screened and selected relevant studies in five subsequent steps (Fig. 1). First, initial search results were uploaded to Covidence, a web-based software for systematic reviews (Veritas Health Innovation, n.d.). Second, once the duplicates were removed, the researchers independently screened all remaining articles by title and abstract. Third, the researchers screened the full-texts of the remaining articles in line with the inclusion and exclusion criteria. Fourth, the researchers screened the reference lists of the included articles. Finally, 10 international experts in the field were consulted for additional articles that did not show up in our search (see the Acknowledgements section for the list of experts). Any disagreement in eligibility was solved either by a) reaching a consensus among two reviewers after discussion or b) discussing it with the last author (LIML). We also calculated a Kappa score to examine the interrater agreement at each stage. Scores between 0–0.20 were considered as none, 0.21–0.39 as minimal, 0.40–0.59 as weak, 0.60–0.79 as moderate, 0.80–0.90 as strong, and above 0.90 as almost perfect agreement between the raters (McHugh, 2012).

### Data extraction

The data extraction was also completed independently by two researchers (TDE and MAT). The researchers extracted data on the descriptive information about the study (e.g., the authors of the study), sample characteristics (e.g., number of participants), descriptive statistics (e.g., mean and standard deviations of the primary outcomes), the type of study (i.e., DD or EMA), measurement tools for the primary outcomes, study characteristics (e.g., number of measurements per day), loss-related factors (e.g., cause of death), and the primary findings of the study. Any disagreement was resolved via discussion.

### Quality and risk of bias assessment

Considering that there is no well-established method to examine the quality and risk of bias for DD or EMA studies (Mölsä et al., 2022), the quality and risk of bias were assessed using the checklist proposed by van Roekel and colleagues (2019). This checklist consisted of items that focused on different aspects of EMA studies, such as the design of the study and compliance. This checklist was also used in previous systematic reviews to investigate the quality and risk of bias in EMA studies (van Dalen et al., 2024). In this systematic review, we omitted the criteria on a) reporting the devices of data collection (e.g., % of participants who use an iOS vs. Android smartphone) because it did not apply to the DD studies, and b) sharing Monte Carlo simulation outputs because it did not apply to any of the included studies. Moreover, we separated the criterion “Scale construction and transformation” into two separate criteria, namely a) scale construction, and b) scale transformation (including centering). Following a similar approach to van Dalen et al. (2024), two researchers (TDE and MAT) independently rated each of the checklist items as “not met’ (0), “met” (1), or not applicable (NA). Then, we converted the rating to percentages, in which higher percentages represent a higher number of applicable criteria met. Following prior research (van Dalen et al., 2024), we qualified rated percentages > 80% as good, between 60% and 80% as sufficient, and < 60% as limited quality.

## Results

### Study selection

The details about the study selection and reasons for exclusion are provided in Fig. 1. Initially, our search yielded 346 records. Four articles were added to the initial list of articles following the screening of the reference lists, and three articles were added after expert consultation, resulting in 353 articles in total. After the removal of duplicates (*k* = 123), 230 unique articles remained for title and abstract screening. Based on this screening, 207 articles were excluded, and the full-texts of the remaining 23 articles were screened, which resulted in the exclusion of three articles due to ineligible study designs (Halberg et al., 1996; Monk et al., 2006; Ryckebosch-Dayez et al., 2016), and another two articles because they did not focus on the daily life after the death of a loved one (Portillo-Van Diest et al., 2024; Saltzman & Hunter, 2024). The interrater reliability Kappa scores were 0.81 for the title and abstract screening and 0.65 for the full-text screening, indicating good and moderate agreement among the researchers, respectively. Discrepancies were solved through consensus. Finally, 18 articles were included in the systematic review.

### Study characteristics

As part of the first aim of our systematic review, we summarize the characteristics of the included studies in Table 2. The studies were conducted either in the United States (*k* = 10), the Netherlands (*k* = 4), Turkey (*k* = 3), or Switzerland (*k* = 1). A DD design was used by 14 of the 18 included studies, while the remaining four studies implemented an EMA design. The duration of the included studies varied between 7 and 98 (*Mdn* = 14) days. The minimum number of measurements during the study was seven, and the maximum number of measurements was 102 (*Mdn* = 20.5). Eight of the studies included bereaved people who experienced the death of a spouse, and three studies included bereaved people who lost a child. The remaining seven studies did not set inclusion criteria regarding the relationship to the deceased. The average time since loss varied between 28 days and 11 years (*Mdn* = 8 months) among the 15 studies that reported how much time, on average, passed since the death of the loved one.

**Table 2:** Overview of the studies (*k* = 18).

Citation	Country of the data collection	Type of study and data collection method	Number of measurements per day/ study duration	Number of bereaved people included, Gender (% women)	Age in years M (SD)	The deceased loved one was a…	Cause of the death	Time since loss, *M* (*SD*)	If included, the population of the comparison group(s)
Aeschlimann et al. (2024)	Switzerland	DD, mobile app	One/ 3 weeks	27, 89%	25.7 (Not reported)	Not reported	Not reported	6 months to 10 years^1^	Not applicable
Bisconti et al. (2004)	United States	DD, paper and pencil	One/ 12 weeks	19, 100%	72.2 (6.1)	Spouse	Not reported	28.8 (7.0) days	Not applicable
Brown et al. (1996)	United States	DD, paper and pencil	One/ Two weeks	94, 79%	55–85 years^1^	Spouse	Not reported	6 months^2^	Non-bereaved adults (*N* = 45)
Buyukcan-Tetik et al. (2024)	Turkey	DD, online survey	One/ One week	483, 47%^3^	Women: 40.4 (10.5) Men: 44.5 (11.1)	Child	Not reported	10.8 (10.0) years	Not applicable
Eisma et al. (2022b)	The Netherlands	DD, paper and pencil	Two/ 10 days	62, 94%^4^	44.0 (14.0)	Partner (45%), Child (15%), Parent (23%), Sibling (6%), Other (11%)	Natural causes (84%) Accident (3%) Murder (2%) Suicide (11%)	22.9 (26.0) months	Not applicable
Ergun et al. (2024)	Turkey	DD, online survey	One/ One week	483, 47%^3^	Women: 40.4 (10.5) Men: 44.5 (11.1)	Child	Not reported	10.8 (10.0) years	Non-bereaved married couples, (*N* = 258 couples, 7 not reported)^3^
Ergun et al. (2025)	The Netherlands	EMA, mobile app	Five/ 14 days	169, 83%	54.2 (12.4)	Partner (48%), Parent (34%), Child (8%), Sibling (4%), Friend (2%), Grandparent (1%), Grandchildren (1%), Other (3%)	Natural cause (80%), Suicide (5%) Accident (5%), Homicide (0%), Other (10%)	20.3 (5.0) weeks	Not applicable
Lenferink et al. (2022)	The Netherlands	EMA, mobile app	Five/ 14 days	80^5^, 78%^6^	41.5 (17.0)	Parent (46%), Grandparent (20%), Partner/spouse (13%), Sibling (5%), Friend (3%), Child (1%), Grandchild (1%,), Other (11%,)	Natural causes (81%), Suicide (8%), Accident (1%), Homicide (%1), Other (%9)	67.8 (90.4) months	Not applicable
Lenferink et al. (2024)	The Netherlands	EMA, mobile app	Five/ 14 days	38, 74%	41.8 (16.5)	Parent (47%), Spouse (13%), Sibling (8%), Grandparent (13%), Friend (5%), Other (13%)	Natural causes (84%), Suicide (8%), Homicide (%3), Other (5%)	6.1 (7.1) years	Not applicable
Mintz et al. (2024)	United States	EMA, mobile app	Six/ 17 days	117, 85%	46.4 (13.9)	Not reported	Not reported	Not reported	Not applicable
Monk et al. (2008)	United States	DD, paper and pencil	One/ 14 days	28, 85%	72.3 (6.4)	Partner	Not reported	8.3 (3.4) months	Not applicable
Monk et al. (2009)	United States	DD, paper and pencil	One/ 14 days	47, 81%	72.3 (7.0)	Spouse	Not reported	9.1 (3.4) months	Older adults with insomnia (*N* = 47), Good sleeper control (*N* = 33)
Monk et al. (2013)	United States	DD, paper and pencil	One/ 14 days	38, 79%	Not reported^7^	Spouse	Not reported	220 (not reported) days^8^	Not applicable
Okun et al. (2011)	United States	DD, paper and pencil	One/ 7 or 14 days^9^	39, 80%	71.6 (7.0)	Not reported	Not reported	Not reported	Good sleeper controls (*N* = 76), Caregivers for spouses with dementia (*N* = 55), Older adults with insomnia (*N* = 52)
Ong et al. (2004)	United States	DD, paper and pencil	One/ 98 days	34, 100%	72.0 (6.1)	Spouse	Not reported	28.6 (6.4) days	Not applicable
Ong et al. (2005)	United States	DD, paper and pencil	One/ 98 days	34, 100%	72.0 (6.1)	Spouse	Not reported	28 (6) days	Not applicable
Ong et al. (2006)	United States	DD, paper and pencil	One/ 98 days	34, 100%	72.0 (6.1)	Spouse	Not reported	28 (6) days	Not applicable
Topal et al. (2025)	Turkey	DD, online survey	One/ One week	483, 47%^3^	Women: 40.4 (10.5) Men: 44.5 (11.1)	Child	Not reported	10.8 (10.0) years	Not applicable

*Note*. DD = Daily diary, EMA = Ecological momentary assessment, *M* = Mean, *SD* = Standard deviation.

1 Only the range was reported.

2 Although the standard deviation was not reported, the authors reported that the range varied from 1 month to 22 months.

3 Some people were included as individuals (without their partners) in the analyses, and their gender/sex was not reported.

4 Only the percentage of women was reported.

5 The value is reported for the full sample. The comparisons between retrospective and momentary recall of prolonged grief symptoms were conducted among 67 people.

6 Only the percentage was reported.

7 Although the sample consisted of people who were older than 60 years old, the mean and standard deviation of age were not reported.

8 The range was from 76 to 498 days.

9 Participants completed the daily surveys either 7 or 14 days, depending on the study protocol. However, the details about the study protocol were not reported in the manuscript.

### Compliance and retention rates

Across the eight DD and four EMA studies that reported the rate of measurement occasions completed, overall compliance rates varied from 55% to 90%. Moreover, overall retention rates (i.e., the rate of people who did not drop out of study participation) varied from 57% to 94%. Notably, the definitions of compliance and retention rates varied across the studies. For instance, while one study included participants only if at least 50% of measurement occasions were completed (Lenferink et al., 2024), another study computed the overall retention rate at 94%, after excluding the participants who withdrew from the EMA (Mintz et al., 2024).

### Quality and risk of bias assessment

**Fig. 2:**
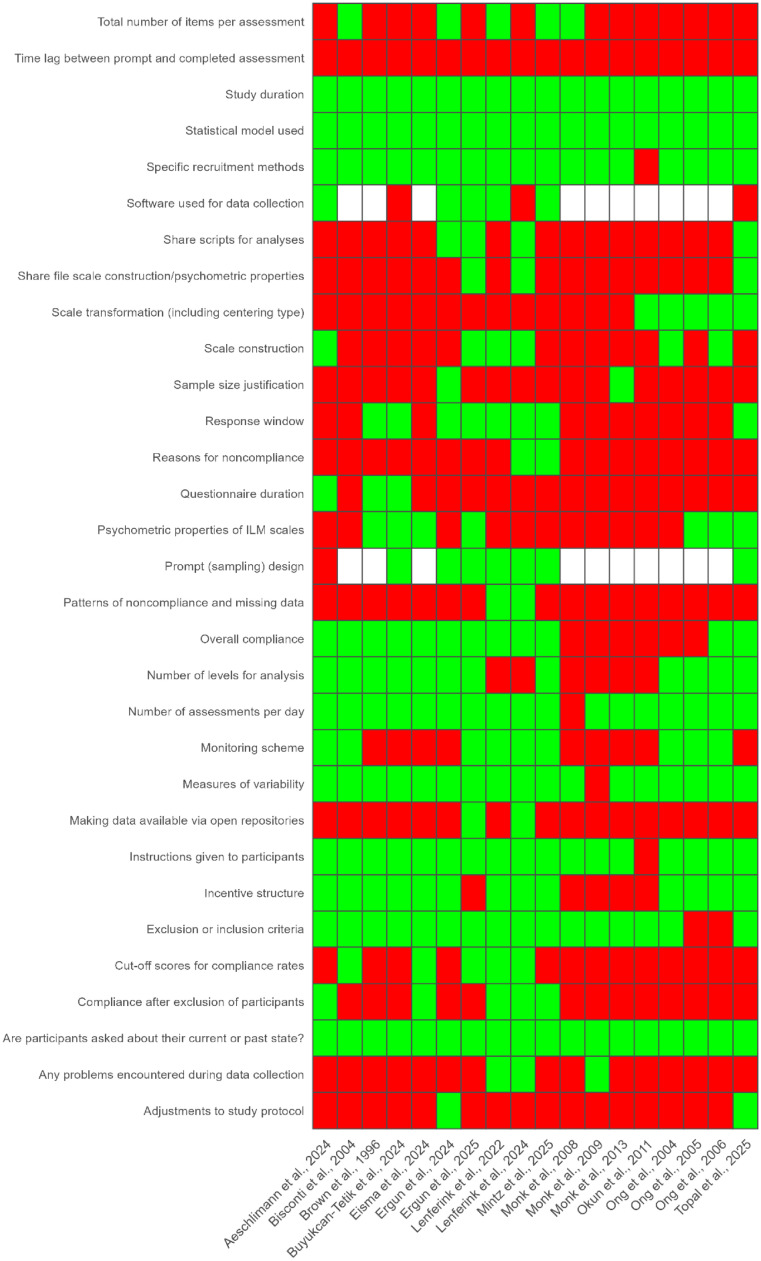
Quality assessment (*k* = 18). *Note*. Green = Criterion was reported, Red = Criterion was not reported, White = Not applicable.

Based on the quality and risk of bias ratings (Fig. 2), none of the studies met the criteria for good quality reporting. While three studies achieved sufficient reporting quality (Ergun et al., 2025; Lenferink et al., 2024, 2022), the reporting of the remaining studies were considered to be of limited quality. The average quality rating was 48%, which varied from 28% to 71%. Three criteria were met by all studies: sufficient reporting on a) study duration, b) if the participants were asked about their current state or past hours/day, and c) statistical models used (please see Fig. 2 for a detailed illustration). In addition, one criterion, namely the time difference between the prompt and the completion of the assessment, was not reported by any of the studies.

### Daily life following the death of a loved one

The second aim of our systematic review was to provide a narrative synthesis of the findings of the included studies regarding the daily life of bereaved people. The 18 studies varied widely in terms of research aims. We identified four main study topics across the studies. Four DD and four EMA studies examined the acceptability, feasibility, and reactivity effects among bereaved people, focusing on the methodological aspects of ILM studies. The remaining DD studies investigated research questions related to the following topics: a) emotions and emotion regulation strategies, b) sleep quality, and c) romantic relationships. The overview of the findings for each topic and the summary of the results are provided in Table 3, Table 4, respectively. For ease of interpretation, we provided the results from the DD studies and EMA studies in different subsections.

**Table 3:** Overview of the studies (*k* = 18).

**Citation of the study**	**The main aim of the study**	**The constructs of interest(s) that was/were measured either using ILM or at baseline (i.e., trait measures) but their associations with ILM measures were examined**	**Instrument(s) used to assess health consequences**
Aeschlimann et al. (2024)	To test the acceptability and feasibility of self-monitoring prolonged grief symptoms via a mobile app	1) Prolonged grief levels 2) Anxiety levels 3) Depression levels 4) Post-traumatic stress disorder levels	1) ICD-11 Prolonged Grief Disorder Scale (IPGDS) 2) Generalized Anxiety Disorder Scale-7 (GAD-7) 3) Patient Health Questionnaire-9 (PHQ-9) 4) International Trauma Questionnaire (ITQ)
Bisconti et al. (2004)	To investigate the pattern of and variability in emotional well-being recently after bereavement	1) Emotional well-being	1) Mental Health Inventory (MHI)
Brown et al. (1996)	To examine differences in social rhythm stability and sleep quality among people who were spousally bereaved and non-bereaved people	1) Social rhythm stability 2) Sleep quality 3) Trait depression levels	1) Social Rhythm Metric (SRM) 2) Pittsburgh Sleep Quality Index (PSQI) 3) Hamilton Rating Scale for Depression
Buyukcan-Tetik et al. (2024)	Investigating the changes in bereaved people’s responses related to mental-health symptoms during a diary study	1) Prolonged grief levels 2) Depression levels 3) Anxiety levels 4) Post-traumatic growth levels	1) Two items: “Today, I felt bitter or angry about the loss of my child” and “Today, I felt that life is meaningless or empty without my lost child”^1^ 2) Two items: “Today, I felt depressed” and “Today, I had very low spirits”^2^ 3) Two items: “Today, I felt very nervous” and “Today, I was anxious and worried” 2 4) Single item: “Today, I thought I could handle difficulties”^3^
Eisma et al. (2022b)	Investigating the daily associations between worry and rumination on the one hand and positive and negative affect on the other hand	1) Daily worry 2) Daily rumination 3) Positive affect 4) Negative affect	1) A single item assessed worry: “To what degree you were worried today” 2) Two single items assessed rumination: “I thought about the reasons for my problems on today” and “I thought about the reasons for my feelings on today.” 3) Negative and positive affect were measured items from aan het Rot and colleagues (2015)
Ergun et al. (2024)	Examining the association between affectionate touch and intimacy among bereaved parents and comparing it to non-bereaved couples	1) Affectionate touch 2) Intimacy	1) Single item: “Today, I touched my partner in an affectionate and caressing manner (e.g., hugged).”^4^ 2) Single item: “Today, I felt close to my partner.”^4^
Ergun et al. (2025)	Investigating the psychometric properties of 11 ESM-PGD items^5^	1) DSM-5-TR prolonged grief symptoms	1) 11 ESM-PGD items^5^
Lenferink et al. (2022)	Testing the acceptability and feasibility of assessing prolonged grief symptoms in daily life	1) DSM-5-TR prolonged grief symptoms 2) Reactions to research participation	1) 11 ESM-PGD items^5^ 2) Personal Benefits and Emotional Reactions subscales of Reactions to Research Participation Scale
Lenferink et al. (2024)	Examining whether prolonged grief symptoms fluctuate within people during an EMA study	1) DSM-5-TR prolonged grief symptoms	1) 11 ESM-PGD items^5^
Mintz et al. (2024)	Testing the acceptability and feasibility of assessing prolonged grief symptoms in daily life	1) Prolonged grief symptoms	1) Single-item questions for different emotions (i.e., yearning, positive emotions, loneliness, anxiety, emotional pain), thoughts in daily life (i.e., thoughts related to the deceased, the death of the loved one, and plans for the future), and behaviors (i.e., activities that make the participants feel closer to the deceased, avoidance of things, leisure activities, and insocial activities)
Monk et al. (2008)	Investigating the sleep quality of widowed people	1) Sleep quality 2) Trait complicated grief levels	1) Pittsburgh Sleep Diary 2) Texas Revised Inventory of Grief
Monk et al. (2009)	Examining the sleep quality of widowed people and comparing it to people with insomnia and people with good sleep quality	1) Sleep quality	1) Pittsburgh Sleep Diary
Monk et al. (2013)	Examining the sleep quality of bereaved people who experienced the death of a spouse	1) Sleep quality 2) Trait depression levels 3) Trait complicated grief levels	1) Pittsburgh Sleep Diary 2) Hamilton Rating Scale for Depression 3) Texas Revised Inventory of Grief
Okun et al. (2011)	Investigating the sleep quality and variability among bereaved people, people with insomnia, caregivers of spouses with dementia, and people with good sleep quality	1) Sleep quality	1) Pittsburgh Sleep Diary
Ong et al. (2004)	Investigating the associations between positive emotions, depression, and anxiety levels of spousally bereaved women	1) Positive emotions 2) Depression levels 3) Anxiety levels 4) Daily stress	1) Subscales of Mental Health Inventory for positive emotions, depression, and anxiety levels 2) A single item, in which participants reported the most stressful event that happened to them throughout the day and how stressful the event was on a 5-point scale
Ong et al. (2005)	Examining the daily associations of perceived control to anxiety and depression among spousally bereaved women	1) Perceived control 2) Anxiety levels 3) Depression levels	1) Behavioral/emotional control, anxiety, and depression subscales of Mental Health Inventory for perceived control, anxiety levels, and depression levels, respectively
Ong et al. (2006)	To investigate the relationships between psychological resilience assessed at baseline, daily positive and negative emotions, and daily stress among women who experienced the death of their spouse	1) Trait psychological resilience 2) Positive emotions 3) Negative emotions 4) Daily stress	1) Dispositional Resilience Scale 2) Subscales of Mental Health Inventory for positive and negative emotions 3) A single item, asking participants the report the most stressful event happened to them on that day and to rate how stressful the event was on a 5-point scale
Topal et al. (2025)	Investigating the dyadic associations between repetitive thought and well-being among bereaved parents	1) Repetitive thought (i.e., yearning and rumination) 2) Individual well-being (Prolonged grief levels and depression symptoms) 3) Relational well-being (Relationship satisfaction, trust, and closeness)	1) Yearning was assessed with two items: “I felt myself longing for my child today”, and “I imagined today what it would be like if my child would still be alive”. Rumination was also assessed with two items: “I thought about the unfairness of my loss today”, and “I thought about how the loss I experienced could have been prevented today”. Both measures was adapted from Eisma and colleagues (2014). 2) Grief levels: Two items, namely “Today, I felt bitter or angry about the loss of my child”, and “Today, I felt that life is meaningless or empty without my lost child”, adapted from TGI-SR (Boelen & Smid, 2017). Depression levels were assessed with two items adapted from Ong and colleagues (2004): “Today, I felt depressed”, and “Today, I had very low spirits”. 3) Relationship satisfaction: A single item, “Today, I was pleased with the relationship with my partner.” Closeness: “Today, I felt close to my partner”. Trust: “Today, I thought my partner was trustworthy”.

1 The items were adapted from the Turkish version of Traumatic Grief Inventory – Self Report Plus (Baş et al., 2022; Boelen et al., 2019).

2 The items were adapted from the study conducted by Ong and colleagues (2004).

3 The items were adapted from the study conducted by Tedeschi and Calhoun (1996).

4 The items were adapted from the study by Debrot and colleagues (2012).

5 Eleven items that tap upon the Prolonged Grief Disorder symptoms according to DSM-5-TR were developed by conducting a cognitive interview with the experts (Lenferink et al., 2022).

**Table 4:** Summary of the studies (*k* = 18).

**Citation of the study**	**Summary of the main findings**
Aeschlimann et al. (2024)	1) People reported lower levels of prolonged grief on average after completing the app compared to baseline levels.
Bisconti et al. (2004)	1) Bereaved people reported increasing levels of emotional well-being over the course of the study. 2) The variability of and fluctuations in bereaved people’s responses decreased over time.
Brown et al. (1996)	1) Social rhythm stability was associated with better sleep quality, however, the association was non-significant when depression levels were controlled for. 2) The social rhythm stability in bereaved people with scores indicating major depression was significantly lower than bereaved people with minor or no depression.
Buyukcan-Tetik et al. (2024)	1) Women’s and men’s grief levels who experienced pregnancy loss decreased during diary participation. 2) There were no changes in the grief levels of parents who experienced the loss during labor or afterwards. 3) Depression levels of neither women nor men changed during the diary. 4) Anxiety levels of women who lost their child during pregnancy and men who experienced the loss during labor or afterwards decreased during the diary. 5) Post-traumatic growth levels of women who lost their child during labor or afterwards decreased during their diary participation.
Eisma et al. (2022b)	1) People reported higher negative affect and lower positive affect when they ruminated more than their own average. 2) Similarly, when people worried more than their own average, they felt higher negative affect and lower positive affect.
Ergun et al. (2024)	1) Bereaved women, but not men, had lower levels of affectionate touch on average than their non-bereaved counterparts. 2) Affectionate touch was positively related to intimacy concurrently both among bereaved and non-bereaved men and women. This positive association was also observed on next day’s intimacy for bereaved women. 3) Rather than the similarity of affectionate touch among partners, both partners’ high levels of affectionate touch contributed to their intimacy.
Ergun et al. (2025)	1) Multilevel confirmatory factor analysis supported one-factor structure of DSM-5-TR PGD symptoms at both between- and within-level. 2) All items fluctuated among people to some extent and were able to differentiate people without and with early elevated PGD symptoms. However, when other psychometric indices (i.e., intraclass correlation coefficients, root-mean square of successive differences, correlations with other mental health-related variables) were considered, not all items were deemed sufficiently to assess prolonged grief in daily life. 3) Based on the abovementioned considerations, two items, namely “In the past three hours, I found myself yearning for him/her.” (i.e., yearning) and “In the past three hours, I felt sad because of his/her death.” (i.e., sadness) formed the Prolonged Grief Symptoms – Short and Ecological Assessment (PGS-SEA) scale to assess prolonged grief in daily life.
Lenferink et al. (2022)	1) On average, participants’ responses to items tapping upon symptoms of yearning, preoccupation with the deceased, marked sense of disbelief, and intense loneliness, were lower when it is momentarily recalled (i.e., during EMA study) compared to retrospective recalling (i.e., during baseline interviews). 2) On average, people reported that participating in the EMA study did not raise any emotional issues for them. However, they reported that they were neutral about whether completing the EMA study was personally beneficial for them.
Lenferink et al. (2024)	1) The item “I felt sad because of his/her death” had the highest fluctuations within people, which was followed by the items “I had intrusive thoughts or images related to the person who died” and “I found myself yearning for him/her”. 2) The highest floor effects were observed for the items “I felt emotionally numb”, “I avoided places, objects, or thoughts that reminded me that he/she is dead”, and “It felt as if a part of me has died along with the deceased.
Mintz et al. (2024)	1) During the EMA study, over time, participants reported lower levels of psychological pain, loneliness, anxiety, thoughts of the deceased, thoughts about the death, and avoiding reminders of the loss.
Monk et al. (2008)	1) Except for the sleep duration, in which it was scored higher among people with lower grief levels, there were no significant differences in sleep quality indicators among people with higher and lower levels of grief assessed at baseline.
Monk et al. (2009)	1) Bereaved people reported worse total sleep time, sleep efficiency, and wake after sleep onset compared to non-bereaved people with good sleep. 2) However, bereaved people also reported better sleep than people with insomnia in the abovementioned indicators.
Monk et al. (2013)	1) Grief levels were negatively associated with time spent asleep. 2) There were no significant associations of sleep efficiency and sleep quality as measured by the Pittsburgh Sleep Quality Index to grief levels.
Okun et al. (2011)	1) On average, the total sleep time was significantly less for bereaved people compared to people with good sleep quality. 2) Bereaved people’s variability in total sleep time and waking time was significantly higher and lower, respectively, compared to people with good sleep quality. 3) On average, bereaved people napped less compared to caregivers of spouses with dementia, older adults with insomnia, and people with good sleep quality.
Ong et al. (2004)	1) People’s anxiety levels were not related to their experiences of positive emotions. 2) The association between daily stress and depression levels were moderated by positive emotions.
Ong et al. (2005)	1) People reported increasing levels of anxiety over the course of the study, which was higher for people who reported higher stress levels assessed at the baseline. 2) There was a positive association between daily stress and anxiety, which was also stronger for people who reported higher baseline stress levels. 3) There was a negative association between daily perceived control and anxiety, and this relationship was stronger among people with higher environmental mastery.
Ong et al. (2006)	1) There were significant positive concurrent and lagged associations between daily stress and negative emotions. Moreover, the lagged association between daily stress and negative emotions was mediated by positive emotions. 2) Trait psychological resilience moderated the association between positive and negative emotions. For people with higher resilience, there was a non-significant concurrent relationship between positive and negative emotion, and this relationship did not differ based on daily stress levels.
Topal et al. (2025)	1) There were no significant within-level associations between repetitive thought and individual well-being. At the between-level, however, repetitive thought grief levels and depression symptoms were negatively associated with individual well-being. 2) Bereaved parents’ more than their own average rumination was associated with their partner’s relational well-being. However, there was no significant lagged association for individual well-being. 3) One’s higher-than-usual rumination was positively associated with their partner’s grief levels among couples who experienced pregnancy loss or people with a shorter time since the death. 4) On average, higher levels of repetitive thought with both one’s own (i.e., actor effect), and their partner’s (i.e., partner effect) individual well-being. This association did not emerge for relational well-being.

*Note*. PGD = Prolonged Grief Disorder, EMA = Ecological Momentary Assessment.

^1^The items were adapted from the Turkish version of Traumatic Grief Inventory – Self Report (Baş et al., 2022; Boelen et al., 2019).

^2^The items were adapted from the study conducted by Ong and colleagues (2004).

^3^The items were adapted from the study conducted by Tedeschi and Calhoun (1996).

^4^The items were adapted from the study by Debrot et al. (2012).

^5^Eleven items that tap upon the PGD symptoms according to DSM-5-TR were developed by conducting a cognitive interview with the experts (Lenferink et al., 2022).

#### DD studies

##### Acceptability, feasibility, and reactivity effects of DD research among bereaved people

Four DD studies examined possible reactivity effects in terms of changes in mental health outcomes. In one of these studies, Aeschlimann and colleagues (2024) tested the acceptability and feasibility of a mobile phone app developed to monitor prolonged grief (PG) symptoms once a day over a period of 21 days. On average, the participants found the mobile app to be acceptable. Moreover, regarding the reactivity effects, on average, participants (*N* = 27) reported lower levels of PG after compared to before using the app. In addition, while in the first two weeks all participants completed the daily measurements, the average compliance dropped to 68% during the third and last week of the study.

In another study, Buyukcan-Tetik and colleagues (2024) studied the changes in mental health outcomes of bereaved parents over the study period (i.e., one week) among 483 bereaved people (228 couples, 27 individuals) who lost a child. The findings of the study indicated that, on average, both men's and women’s PG levels decreased across 7 days, while depression symptoms did not change, indicating a potential positive reactivity effect for PG levels. However, subgroup analyses revealed that the change in PG levels occurred only in parents who experienced pregnancy loss, but there was no significant change in PG levels in parents who experienced child loss during labour or afterwards. In addition, women who experienced pregnancy loss and men who experienced child loss during or after labor showed a decrease in anxiety symptoms during the diary. Lastly, women who lost their child during labor or afterwards experienced a decline in their post-traumatic growth levels during their participation in the DD study.

Moreover, two other DD studies have revealed findings related to the reactivity effects of DD studies. In one of these studies, Bisconti and colleagues (2004) found that over the course of the study period (i.e., 12 weeks), the daily emotional well-being of 19 people who experienced the death of a spouse increased, which suggests a potential positive reactivity effect. However, in another study using the data from the same larger research project, Ong and colleagues (2005) found that over the 98-day study period, anxiety levels of 34 spousally bereaved women increased, and this increase was steeper for participants who reported higher levels of trait stress at baseline. Both studies are further discussed in detail under the *Emotions and emotion regulation strategies* section.

Overall, these studies showed that DD is an acceptable and feasible method to assess grief in daily life. Regarding reactivity effects, the findings from two studies suggested that bereaved people’s PG levels decreased, both when comparing PG levels from before to after completing diaries and over the study period. However, one study reported increasing levels of anxiety, which was moderated by their trait stress levels (Ong et al., 2005). This suggests that participating in DD studies is not harmful, because it does not decrease mental health outcomes on average. Instead, it could potentially benefit bereaved people by alleviating their distress.

##### Emotions and emotion regulation strategies

Six DD studies examined the experiences of positive emotions and/or emotion regulation strategies in bereaved people. In one of these studies, Bisconti and colleagues (2004) investigated the fluctuations and course of daily emotional well-being during the study period (i.e., 12 weeks) among 19 spousally bereaved women. The initial fluctuations in bereaved people’s responses at baseline did not predict the overall course of their emotional well-being across 12 weeks. In addition, over the course of the study, the emotional well-being of bereaved people showed a damping pattern, indicating that fluctuations in bereaved people’s emotional well-being decreased over time.

Using a subset of data from the same larger research project, Ong and colleagues (2004) investigated how positive emotions and humor as a coping strategy are associated with depression, anxiety, and stress in daily life among a community sample of 34 women whose spouse died. They found a significant negative association between state positive emotions and state depression levels. Moreover, positive emotions moderated the association between state stress and depression symptoms, indicating that the relationship between state stress and state depression symptoms was weaker when participants experienced higher positive emotions. Lastly, aggregated levels of positive emotions were not related to people’s state anxiety levels.

In another study using the data from the same project, Ong and colleagues (2005) investigated the associations between daily perceived control, depression, anxiety, and stress among 34 spousally bereaved women. They found that over the 98-day study period, anxiety levels increased, and this increase was steeper for participants who reported higher levels of trait stress at baseline. In addition, daily stress was positively related to daily anxiety. Moreover, higher levels of daily perceived control were associated with lower levels of anxiety, and this association was stronger for people with higher levels of environmental mastery assessed at baseline.

Again, using the same project data, Ong and colleagues (2006) investigated the concurrent and temporal relationships between trait resilience, daily stress, and daily positive and negative emotions. Daily stress had a positive concurrent and lagged relationship with negative emotions. Furthermore, the lagged association was mediated by daily positive emotions. In addition, daily positive and negative emotions were negatively related, and this association was moderated by trait resilience levels. In other words, daily stress was positively related to daily negative emotions on the same day and the next day, and the lack of positive emotions mediated the association between daily stress and the next day’s negative emotions. Moreover, for people who reported low trait resilience, there was a negative concurrent relationship between positive emotions and negative emotions, which was stronger on days with higher levels of stress. However, for highly resilient people, the relationship between positive and negative emotions was non-significant, and did not depend on the perceived stress of the day.

Another study explored bereaved people’s emotion regulation strategies in daily life. Eisma et al. (2022b) investigated if and to what extent both trait and state rumination and worry were associated with state positive and negative affect in a community sample of 62 bereaved people. They found that people who reported higher trait-levels of rumination and worry reported more state negative affect and less state positive affect on average. Moreover, PG levels at baseline significantly moderated the relationship between state rumination and negative affect, such that the positive association between state rumination and negative affect was stronger among people with higher (vs. lower) PG levels. However, none of the other associations between trait and state rumination and trait and state positive and negative affect were moderated by PG intensity assessed at baseline. Lastly, on days when people reported higher state rumination and worry levels than their own average, they also reported higher negative affect and lower positive affect state levels. Overall, while trait rumination and worry were positively related to state negative affect and negatively to state positive affect, PG levels only moderated the relationship between state rumination and negative affect.

Lastly, Topal and colleagues (2025), using the same dataset as Buyukcan-Tetik et al. (2024), examined to what extent repetitive thought (i.e., yearning and rumination) were related to individual (i.e., PG levels and depression symptoms) and relational (i.e., relationship satisfaction, closeness, and trust) well-being among 483 bereaved people. Their findings revealed that bereaved parents’ overall repetitive thought were negatively associated with both their own and their partner’s individual well-being. However, there were no significant bidirectional lagged within-person associations between repetitive thought and individual well-being.

Taken together, various emotions and emotion regulation strategies have been studied in relation to bereaved people’s daily mental health. In general, maladaptive emotion regulations strategies such as worry and rumination, were linked to poorer mental health, while positive emotions, such as daily perceived control and trait resilience, were linked to better mental health.

##### Sleep quality

Five DD studies investigated the sleep quality of bereaved people. In one of these studies, Brown and colleagues (1996) investigated bereaved people’s sleep quality and social rhythm stability (SRS), which refers to the stability of the timeline of habitual events that occur on a daily basis, such as getting out of bed or having dinner. Participants were categorized into three groups based on their depression levels: syndromal major depression (*n* = 44), minor depression (*n* = 26), and no depression (*n* = 24). SRS was positively associated with better subjective sleep quality, but the association became non-significant when depression was controlled for. Moreover, the SRS was negatively related with depression scores assessed at baseline in the total sample, and people with major depression had a lower SRS than people in the two other groups.

Similarly, Monk and colleagues (2008) investigated the sleep quality and SRS in a community spousal bereaved sample (*N* = 28). They found no significant association between CG levels of bereaved people and their SRS. In addition, CG levels were not associated with any of the sleep-related variables measured during the study period. Moreover, there were no moderation effects by the expectedness of the death on these non-significant associations.

In another study, Monk and colleagues (2009) compared the sleep quality of 47 bereaved people whose spouse died to 47 non-bereaved older adults who met insomnia disorder criteria according to DSM-IV (i.e., older adults with insomnia) and 33 non-bereaved adults who did not meet the insomnia disorder criteria (i.e., good sleeper controls). Their findings revealed that spousal bereaved people reported better sleep than older adults with insomnia, as indicated by total sleep time, sleep efficiency, and time spent awake after starting to sleep, but worse quality of sleep compared to good sleeper controls.

Relatedly, Okun and colleagues (2011) compared the sleep quality among four groups: a) 39 bereaved adults who reported poor sleep quality as indicated by the Pittsburgh Sleep Quality Index (PSQI; Buysse et al., 1989), b) caregivers of spouses with dementia (*n* = 55), c) older adults with insomnia (*n* = 52), and d) good sleeper controls (*n* = 76). Their findings revealed that, on average, bereaved people had shorter total sleep time than good sleeper controls. Moreover, compared to good sleeper controls, bereaved adults showed significantly greater variability in waking time and significantly less variability in total sleep time across the study period. Lastly, bereaved people reported taking less frequent naps in daily life compared to dementia caregivers, older adults with insomnia and good sleeper controls.

Lastly, one study included 38 people from a community spousal bereaved sample (Monk et al., 2013). The findings revealed that widow(er)s who had higher trait depression levels reported worse sleep quality compared to widow(er)s with lower trait depression levels. Complicated grief (CG) levels were negatively associated with time spent asleep. However, CG levels were not related to sleep efficiency, which was quantified as the rate of time spent asleep to the total time spent in bed, and sleep quality. In addition, the time since the loss was not predictive of daily sleep quality.

Overall, mixed findings were found regarding the association between CG and sleep quality. Some studies found that CG was inversely related to sleep, while some studies found no association. More research is needed to disentangle if and how mental health outcomes are related to sleep in daily life after loss.

##### Romantic relationships

The study conducted by Topal and colleagues (2025) also investigated the romantic relationships of bereaved people who experienced child loss before, during, or after labor. Their findings on the within-person associations revealed that there was a negative lagged association between one’s increases in rumination and their partner’s relational well-being the next day. Further post-hoc analyses on the negative association between rumination and relational well-being suggested that this association was observed among bereaved people who had a shorter time since loss or experienced pregnancy loss. However, regarding the between-person associations, there were no significant associations between repetitive thought and relational well-being.

Another study using the same dataset as Buyukcan-Tetik and colleagues (2024) and Topal and colleagues (2025), Ergun and colleagues (2024) investigated affectionate touch and its associations with feelings of intimacy among 483 bereaved parents and compared the findings with 523 non-bereaved married partners (258 couples, seven individuals) who did not experience child loss. On average, bereaved women reported lower levels of affectionate touch compared to non-bereaved women, while this discrepancy did not emerge among bereaved and non-bereaved men. Among both bereaved and non-bereaved couples, affectionate touch was positively related to feelings of intimacy on the same day, while this positive influence extended to the next day’s intimacy for bereaved women. Lastly, findings revealed that if both partners of a couple reported higher affectionate touch levels, they reported higher levels of intimacy.

Taken together, the findings from the abovementioned studies highlight that the experiences and grief reactions of bereaved parents who lost a child are interdependent and also unfold in the parents’ romantic relationships. Moreover, the mental health of bereaved parents could also influence their romantic relationships, and some romantic relationship behaviors in daily life, such as affectionate touch, could benefit bereaved couples’ daily relationship functioning. However, more research is warranted to draw firm conclusions regarding the daily relational impact of the death of a loved one.

#### EMA studies

##### Acceptability, feasibility, and reactivity effects of EMA research among bereaved people

Four studies examined whether EMA is an acceptable and feasible method to assess the daily life experiences of bereaved people. Lenferink and colleagues (2022) investigated whether EMA is a feasible and acceptable data collection method to assess PG reactions in daily life in a community bereaved sample of 80 adults, and to what extent momentarily reported PGD symptoms differ from retrospectively recalled symptoms. On average, people reported lower PG levels after the EMA phase compared to the baseline levels, which could be considered an indicator of a reactivity effect. The results revealed that overall compliance was moderate (60%). Regarding emotional reactions to intensive data collection, most people were neutral about whether repeatedly answering questions on PG in daily life was personally beneficial and reported that participating in the EMA study did not raise any emotional issues. Moreover, for four PG symptoms, namely yearning, preoccupation with the deceased, marked sense of disbelief, and intense loneliness, the momentary ratings were significantly lower than retrospectively recalled ratings. No significant differences were found for the remaining seven PG symptoms.

Similarly, Mintz and colleagues (2024) conducted an EMA study including 117 bereaved people who had been undergoing PG treatment for the last two months to test the feasibility, acceptability, and reactivity of an EMA design. Regarding feasibility, overall compliance was relatively high (94%) when participants who withdrew from the EMA study were excluded. Moreover, people on average found participating in the EMA study easy and not distressing or burdening, while they believed their responses contributed to scientific knowledge, suggesting that EMA is an acceptable method to assess PG in daily life. Regarding potential measurement reactivity, while some PG symptoms, namely psychological pain, loneliness, anxiety, thoughts of the deceased and death, and avoidance, slightly improved during the EMA participation, the other symptoms, such as yearning and positive emotions, did not change over time.

Lenferink et al. (2024) examined to what extent 38 bereaved people’s PG symptoms fluctuated during their participation in an EMA study, quantified by the root mean square of successive differences (RMSSD) scores, using the same study sample as in Lenferink et al. (2022). The compliance rate was nearly 79%, while the retention rate was 68%. All items assessing PG symptoms seemed to fluctuate to some extent. The greatest fluctuations, as indicated by RMSSD scores, were observed for the items assessing sadness because of the loss (*M* = 0.76, *SD* = 0.57), having intrusive thoughts related to the deceased or loss (*M* = 0.66, *SD* = 0.60), and yearning for the deceased (*M* = 0.58, *SD* = 0.48). In addition, items assessing emotional numbness, avoidance of reminders, and feeling as if a part of the self has died along with the deceased, showed the highest floor effects (i.e., some participants reported not experiencing these symptoms at any measurement occasion).

Lastly, Ergun et al. (2025) investigated the psychometric properties of the 11 items that were developed by Lenferink and colleagues (2022) to assess PG symptoms in daily life. To do so, they evaluated their measurement structure, between- and within-person reliability, and the presence and extent of short-term fluctuations of the symptoms among 169 people bereaved three to six months earlier. The compliance was moderate (71%). While the findings supported the hypothesized one-factor model of PG symptoms between and within people, not all items performed equally well in capturing between-person differences and within-person variations of PG. Based on these findings, EMA was considered to be an acceptable and feasible method to assess bereaved people’s everyday functioning in daily life and to capture the dynamic nature of bereavement. However, some symptom items (e.g., yearning) appeared more relevant than others (e.g., avoidance) for EMA studies. A Prolonged Grief Symptoms - Short and Ecological Assessment (PGS-SEA) scale, which includes items capturing the yearning and sadness over the loss symptoms, was suggested for use in future EMA research.

Overall, the few EMA studies that have been conducted among bereaved people indicate that it is an acceptable research method based on participants’ feedback regarding EMA participation. Regarding feasibility, it seems that the overall compliance rates are moderate to high. When comparing mental health levels from before to after EMA research, we tentatively conclude that participating seems to coincide with reduction in overall mental health levels.

## Discussion

In this systematic review, we aimed to provide an overview of the characteristics, reporting quality, and findings of ILM studies investigating daily life dynamics in psychological, social, and physical functioning among bereaved people. Eighteen articles were identified and reviewed that examined different methodological questions related to acceptability, feasibility, and reactivity effects of ILM among bereaved people, and theoretical questions concerning emotions and emotion regulation strategies, sleep quality, and romantic relationships.

### Acceptability, feasibility, and reactivity effects of ILM among bereaved people

The acceptability, feasibility, and reactivity effects of ILM among bereaved people were investigated across four DD and four EMA studies. Because PGD was only recently introduced as a mental disorder in the ICD and DSM systems, and earlier studies did not use EMA to examine PG in daily life, it is not unexpected that a substantial part of the identified EMA studies focused on the acceptability and feasibility of EMA in this target group. Despite the variability in the methods (i.e., DD and EMA) and measures used to assess grief levels across studies, findings from all studies supported ILM as an acceptable and feasible method to assess mental health symptoms in daily life after loss. Moreover, earlier theoretical frameworks have suggested that the dynamic nature of grief is part of a non-disturbed grieving process (Stroebe & Schut, 1999). Although the abovementioned studies have focused on PG symptoms, which indicate intense and persistent distress following bereavement, the findings could suggest that the fluctuations might also be part of a disturbed grieving process. This also highlights the relevance and potential of using ILM to investigate both non-disturbed grief and PG in daily life.

### DD studies

#### Emotions and emotion regulation strategies

Commonly studied topics in DD studies were daily emotions and emotion regulation strategies of bereaved people. The results from these studies supported previous cross-sectional findings suggesting that maladaptive emotion regulation, such as worry and rumination, was associated with higher PG levels (Eisma & Stroebe, 2021). In addition, these findings align with cognitive-behavioural theories about grief postulating the crucial role of negative thinking in the onset and maintenance of PGD (Boelen et al., 2006), and also suggest that the emotions and emotion regulation strategies are not stable in daily life, which warrants a dynamic research approach. Moreover, regarding the findings on the benefits of positive emotions, broaden-and-build theory suggests that the experiences of positive emotions could be a source of resilience and promote individual well-being and satisfaction with life (Fredrickson, 2004). Therefore, it is possible that the daily experiences of positive emotions help bereaved people cope with their grief reactions and buffer the emotional distress caused by the death of a loved one (Boelen & Lenferink, 2020; Tweed & Tweed, 2011). When considered together, these studies highlight the negative impact of maladaptive emotion regulation strategies and the positive impact of positive emotions, aligning with theoretical models that introduce them as barriers and resources for mental health, respectively (Bohlmeijer & Westerhof, 2021).

#### Sleep quality

The sleep quality of bereaved people was investigated by five DD studies. In their systematic review, including mostly cross-sectional studies but also longitudinal studies, Lancel and colleagues (2020) highlighted the role of bereavement in sleep disturbances and impairments, and vice versa. Despite this, the ILM studies in the current review revealed mostly non-significant findings regarding the associations between mental health indicators (e.g., grief levels) and daily sleep quality. Considering that one of the DD studies did reveal a negative association between depression symptoms and sleep quality (Monk et al., 2013), other risk factors – such as depression – could potentially explain how the death of a loved one affects bereaved people’s daily sleep quality (Lancel et al., 2020). Moreover, the discrepancy between the negative results in cross-sectional designs and non-significant findings in ILM studies may highlight the added value of ILM in assessing sleep quality, as momentary assessments could capture the disturbances in sleep more accurately and in an ecologically valid manner (Scollon et al., 2003).

#### Romantic relationships

Related to social functioning, two DD studies focused on the romantic relationships of bereaved couples who experienced the death of their child. The studies found that there was a negative lagged association between one's rumination and partner's relational well-being (Topal et al., 2025), and affectionate touch was positively associated with intimacy among bereaved couples (Ergun et al., 2024). These findings align with previous studies indicating that maladaptive emotion regulation strategies could harm romantic relationships (Caldwell et al., 2019), and extend prior research on the benefits of affectionate touch on intimacy among non-distressed couples (Debrot et al., 2013; Durbin et al., 2021; Jakubiak & Feeney, 2017) to bereaved couples. Moreover, these results suggest that the daily experiences of bereaved couples are interconnected to each other, and vary depending on the dynamics of their romantic relationships. For instance, while repetitive thought could be a risk factor that interferes with daily relationship functioning, affectionate touch could be a protective factor which could buffer the negative impact of the death of a loved one on romantic relationships. Therefore, future ILM studies could consider taking the role of social relationships in coping with bereavement into account and incorporate dyadic methods that provide an opportunity to investigate how bereavement unfolds in different types of close relationships, such as parent-child dyads and romantic couples.

### Quality assessment

Assessing the reporting quality of the studies, we conclude that none of the studies met the criteria for good quality of reporting. Only three studies (Ergun et al., 2025; Lenferink et al., 2024, 2022) were considered to be of sufficient quality. While one criterion, that is, the time difference between the prompt and completed assessment, was not reported in any of the studies, some other criteria, such as the study duration and whether the participants were asked about their current or past states, were reported consistently across all studies. However, more detailed and comprehensive reporting of study designs is essential to draw reliable and valid conclusions. Therefore, future research could report more detailed information regarding their study design and procedure. Below, we provide an overview of the state of the field and formulate some recommendations for future research.

### State of the field and recommendations for future research

The included ILM studies provided valuable insights into different aspects of daily life after the death of a loved one. As the third aim of our study, we identified several common characteristics of the included studies that could be improved to increase their interpretability and formulated some suggestions and directions for future ILM research.

#### Providing more information about the study design

The quality assessment of the studies included in our review showed that some essential ILM quality criteria, such as sample size justification, compliance, and retention rates, were not consistently reported. Systematic and comprehensive reporting on these aspects of the studies would not only improve the transparency of the specific study but also enable the researchers to learn more about the practices of the studies conducted and consider possible factors, such as the cause of death, that could influence bereaved people’s participation tendencies. Therefore, we suggest that future researchers provide more detailed information about their study designs and protocols, if possible, by following guidelines that are suitable for their research design and question (e.g., Liao et al., 2016; Trull & Ebner-Priemer, 2020).

#### Detailed reporting about and diversity in sample characteristics

Another shared aspect of the included studies was that the study samples consisted mostly of community samples (except for Mintz et al., 2024). Therefore, whether the findings of these studies also generalize to bereaved people who are highly distressed and seeking treatment remains limited. It is possible that people with elevated levels of distress have also adopted maladaptive coping mechanisms, such as worry and rumination, more than bereaved people with lower levels of distress (Eisma et al., 2022a; Eisma & Stroebe, 2021), which shapes their daily life experiences. In addition, some other death-related factors, such as a traumatic death, could affect the daily life of bereaved people more intensely due to the complex grieving processes (e.g., Boelen et al., 2015; Kauffman, 2013), which could be investigated by future studies. Considering that only one of the reviewed studies consisted of a treatment-seeking sample, further research is needed to understand to what extent and how the death of a loved one unfolds in the daily lives of bereaved people with higher distress levels.

Moreover, there was a wide variation in the levels of detail about the death-related factors that were reported by the researchers. For instance, factors such as the time passed since the death or the cause of death (Buur et al., 2024), which have been shown to be related to PG levels, were not measured or reported consistently across all studies. In addition, there were some sample characteristics, such as the gender of participants and kinship to the deceased, that showed limited diversity across studies. For instance, the majority of the study participants were women, which is also a common pattern among PG research (Buur et al., 2024). It is likely that these loss-related factors and sample characteristics also affect bereaved people’s daily life experiences following the death of a loved one. Therefore, we encourage future researchers to assess and provide more information about the factors and characteristics that are potentially related to the everyday experiences of bereaved people.

Lastly, another common feature of the included studies was the limited variability in the countries of data collection, namely in the United States (*k* = 10), the Netherlands (*k* = 4), Turkey (*k* = 3), or Switzerland (*k* = 1). However, how grief experience may vary across different cultures and countries (Rosenblatt, 2017; Stroebe & Schut, 1998). For instance, a cross-sectional study conducted by Mazza and colleagues (2024) revealed that the centrality of yearning, which is conceptualized to be one of the key symptoms of PGD, varies across different cultures. Because the lack of variation in the country of data collection limits the generalizability of the results, we recommend future research to investigate the everyday lives of bereaved people by conducting ILM studies in different cultures and countries.

#### Considerations about the study design

Only four of the reviewed studies used EMA designs, while the remaining 14 studies used DD designs. Although DD designs provide valuable information regarding the day-to-day changes and experiences of bereaved people (e.g., related to sleep quality), they may lack the granularity to capture moment-to-moment fluctuations in experiences, behaviours and contexts within everyday life as effectively as EMA designs can (Myin-Germeys & Kuppens, 2022). Hence, EMA studies offer a unique opportunity to investigate a wide array of research questions in different daily life aspects momentarily and in more detail, such as whether people report lower levels of PG if they perceive their partner to be responsive towards them, or examining lagged associations between momentary levels of social support and PG. Moreover, the initial EMA studies supported the notion that grief reactions are indeed dynamic and fluctuate within bereaved people’s everyday lives (Ergun et al., 2025; Lenferink et al., 2024). Therefore, future studies may consider implementing fine-grained EMA assessments rather than a DD, when it is empirically and/or theoretically justified, to investigate the daily life following the death of a loved one.

Moreover, most of the included studies used measures that were validated for cross-sectional studies, but not for ILM studies. However, measures used in ILM may require different psychometric approaches and testing methods compared to those used for cross-sectional measures (Dejonckheere et al., 2022; Eisele et al., 2024; Myin-Germeys & Kuppens, 2022). For instance, studies on the development and psychometric evaluation of items to assess PG in daily life have revealed that some items used in cross-sectional measures may not be sufficiently able to capture the daily life dynamics (Ergun et al., 2025; Lenferink et al., 2022). Hence, we recommend researchers to thoroughly test and report the psychometric properties of the items used in their ILM studies, and use previously validated ILM measures whenever possible.

Relatedly, it is recommended by experts in the trauma research field that studies make their data as Findable, Accessible, Interoperable, and Reusable (FAIR) as possible (see Hruska et al., 2025; Pociūnaitė-Ott & Lenferink, 2025, for detailed discussion). The FAIR principles include but are not limited to describing the data and metadata in detail, posting codebooks that include explanations about the study variables to a trusted repository, the data and other materials are linked with each other, and providing instructions on using the data in the future. Because these principles not only allow researchers to share and reuse existing datasets, but also contribute to the advancement of science and promote the transparency of their research, we recommend researchers to follow FAIR principles as much as possible.

### Limitations of the current review

Our systematic review had several limitations that should be acknowledged. First, although most of the included studies concerned DD designs, the quality assessment tool used in our systematic review was developed to assess the quality of EMA studies for adolescent samples specifically. Second, our decision to search for articles only in English could have limited the number of studies in our review. Lastly, we did not include any book chapters or unpublished work (e.g., unpublished PhD dissertations) that may have investigated the daily life impact of the death of a loved one. Moreover, we were unable to review the most recent ILM studies that were published after our search in May 2025 (Franzen & Lenferink, 2025; Kivelä et al., 2026; Marcolini et al., 2026; Pociūnaitė-Ott et al., 2026, 2025; Specker et al., 2025; Stelzer et al., 2025; Zhou et al., 2025). Lastly, we did not include any book chapters or unpublished work (e.g., unpublished PhD dissertations) that may have investigated the daily life impact of the death of a loved one.

## Conclusion

This review shows that DD and EMA are acceptable and feasible methods to examine bereaved people’s daily life experiences. Despite the low number of studies included, the variation in the topics of the studies highlights that the death of a loved one can have a multifaceted impact on bereaved people’s everyday lives, including sleep quality, emotion-regulation strategies, and romantic relationships. Considering the rapid growth and advantages of ILM in mental health research and the dynamic nature of bereavement, we encourage bereavement researchers to implement ILM more frequently in their studies to capture everyday experiences of bereavement.

## Declaration of generative AI and AI-assisted technologies in the manuscript preparation process

During the preparation of this work, the author(s) have used ChatGPT and Grammarly in order to check the grammar and improve the readability and language of the manuscript. After using these tools, the author(s) reviewed and edited the content as needed and take(s) full responsibility for the content of the published article.

## Funding

This publication is funded by the University of Twente and is part of the project 'Toward personalised bereavement care: Examining individual differences in response to grief treatment’ [ID: Vl.Veni. 211 G.065] of the research programme [NWO Talent Programme 2021 -Veni], which is financed by the Dutch Research Council (NWO) and awarded to Lonneke I.M. Lenferink.

## CRediT authorship contribution statement

**Turan Deniz Ergun:** Conceptualization, Methodology, Formal analysis, Investigation, Data curation, Writing – original draft. **Mustafa Anil Topal:** Formal analysis, Investigation, Writing – review & editing. **Peter M. ten Klooster:** Conceptualization, Writing – review & editing, Supervision. **Gerben J. Westerhof:** Conceptualization, Writing – review & editing, Supervision. **Ernst T. Bohlmeijer:** Conceptualization, Writing – review & editing, Supervision. **Lonneke I.M. Lenferink:** Conceptualization, Supervision, Writing – review & editing, Funding acquisition.

## Declaration of competing interest

The authors declare that they have no known competing financial interests or personal relationships that could have appeared to influence the work reported in this paper.
